# LLC tumor cells-derivated factors reduces adipogenesis in co-culture system

**DOI:** 10.1016/j.heliyon.2018.e00708

**Published:** 2018-07-30

**Authors:** Magno Alves Lopes, Felipe Oliveira Franco, Felipe Henriques, Sidney Barnabé Peres, Miguel Luiz Batista

**Affiliations:** a Laboratory of Adipose Tissue Biology, Center for Integrated Biotechnology, University of Mogi das Cruzes, Mogi das Cruzes, São Paulo, Brazil; b Program in Molecular Medicine, University of Massachusetts Medical School, Worcester, Massachusetts, USA; c Department of Physiological Sciences, State University of Maringá, Maringá, Paraná, Brazil

**Keywords:** Cancer research, Cell biology, Molecular biology

## Abstract

Cancer cachexia (CC) is a multifactorial syndrome with an unknown etiology. The primary symptom is the progressive reduction of the body weight. Recently, down-regulation of adipogenic and lipogenic genes were demonstrated to be early affected during cachexia progression in adipose tissue (AT), resulting in AT remodeling. Thus, this study aimed to evaluate in a co-culture system the influence of the Lewis Lung Carcinoma (LLC) tumor cells (c/c-LLC) in an established pre-adipocyte cell line 3T3-L1 adipogenic capacity. c/c-LLC in the presence of 3T3-L1 caused a reduction in lipids accumulation, suggesting that secretory tumor cells products may affect adipogenesis. Interestingly, a very early (day 2) down-regulation of proliferator-activated receptor gamma (PPARγ) and CCAAT/enhancer-binding protein alpha (C/EBPα), followed by late genes (day 4 and 8), adiponectin, perilipin, and fatty acid-binding protein 4 (FABP4). Caspase-3 expression was increased on the last day of cell differentiation; it occurred in the expression of pro-inflammatory cytokines interleukin-6 (IL-6) and tumor necrosis factor alpha (TNF-α). Overall, our results suggest that LLC secretory products impair adipocyte differentiation in a co-culture system and increased apoptosis. In summary, our study has shown the inhibition of the adipogenic process in the 3T3-L1 co-culture system with LLC cells.

## 1 Introduction

Cancer cachexia (CC) is described as a complex, multifactorial syndrome with a difficult treatment that directly impacts in the patient's quality of life (Fearon et al., 2012; Tsoli and Robertson, 2013; Blum et al., 2014). The main symptoms include involuntary and progressive body weight loss, anorexia, disruption of energy metabolism, systemic inflammation and deficiency in immune system function, which makes CC responsible for the deaths of 22–40% of cancer patients (Argiles et al., 2003; Tsoli et al., 2014; Seelaender et al., 2015; Batista et al., 2016). During the development of CC, many organs are affected, such as liver, brain, immune system, heart, skeletal muscle and adipose tissue (AT) (Batista et al., 2012a; Porporato, 2016). The depletion of fat mass occurs faster than the observed in skeletal muscle, which suggests that AT is affected early during the syndrome (Fouladiun et al., 2005; Das et al., 2011). A conceivable explanation is the down-regulation of pro-adipogenic transcription factors, *i.e.,* peroxisome proliferator-activated receptor gamma (PPARγ) and CCAAT/enhancer-binding protein alpha (C/EBPα), phenotypic proteins, i.e., perilipin, fatty acid-binding protein 4 (FABP4), triglyceride lipase (ATGL) and hormone-sensitive lipase (HSL) (Bing and Trayhurn, 2009) directly influenced by the production of inflammatory cytokines due to immune cell infiltration (Henriques et al., 2017). Those are two significant events during the AT remodeling induced by cancer cachexia (Batista et al., 2016; Franco et al., 2017). AT atrophy and its consequent remodeling is worsened by the due to increased lipolysis and decreased lipogenesis in adipocytes (Ryden et al., 2008; Batista et al., 2012a; Henriques et al., 2017). Impairment in the balance between the preadipocytes that entering in adipogenesis process and mature adipocytes that are undergoing apoptosis, a process known as cell turnover has been recently demonstrated in AT from an animal model of cancer cachexia (Arner and Spalding, 2010; Franco et al., 2017).

In addition, several studies have emphasized the relationship between weight loss, with a high concentration of inflammatory cytokines present in serum and tissue of animal models and CC patients, such as interleukin-6 (IL-6), tumor necrosis factor alpha (TNF-α) (Tsoli and Robertson, 2013; Argiles et al., 2014; Camargo et al., 2015). Both adipocytes and tumor cells can secrete pro-inflammatory cytokines, which together establish several different functions, such as cell cycle control, apoptosis signals, and control of the hormones expression that regulate energy balance (Trayhurn and Wood, 2004; Camargo et al., 2015). Inflammatory signals from tumors intersect with the normal cross-talk between adipose tissue and other organs, leading to impaired energy balance and catabolism of fat and muscle (Tsoli and Robertson, 2013; Seelaender et al., 2015). In the same way, in the setting of cancer cachexia, IL-6 and TNF-α are considered to be critical drivers of lipolysis and fat depletion, with recent evidence of AT production of these cytokines. Such scenario is followed by increased recruitment of inflammatory cells that could account for the elevated circulating levels of IL-6 observed in CC patients (Batista et al., 2012b, 2013, 2016) and animal model (Bing et al., 2006; Tsoli and Robertson, 2013).

It has been previously reported that pro adipogenic transcription factors as PPARγ and C/EBPα, are downregulated in the initial stage of the CC syndrome in visceral fat depots, even before the local AT and systemic inflammation installation (Batista et al., 2012a). This downregulation suggests that other factors may be involved in reducing expression of these genes, at least during the early stages of the disease. However, this phenomenon was not studied at cellular levels using in vitro model. In this study, we evaluated whether co-culture with Lewis Lung Carcinoma (LLC) tumor cells *per se* may influence the adipogenesis process, in particular addressing the production of inflammatory cytokines by both tumor and 3T3-L1 cells.

## 2 Materials and methods

### 2.1 Cell culture

Swiss preadipocytes 3T3-L1 cell line (ATCC^®^ CL173™ - American Type Culture Collection, Manassas, VA, USA), were plated at 1 × 10^4^ in 24-well culture plates and cultured in Dulbecco's Modified Eagle's Medium (DMEM) high glucose with L-glutamine (4 mM) (Gibco, Carlsbad, CA, USA, Cat. No. 11965084), supplemented with 10% bovine serum (Gibco, Cat. No. 16170) and 2% penicillin with streptomycin (Gibco, Cat. No. 15140122) at pH 7.4. The cells were maintained at 37 °C with 5% carbon dioxide (CO_2_) so as not to reach complete confluence until they were induced to differentiate (Ailhaud, 2001). Preadipocytes were brought to complete confluence (day -2) and after two days of confluence (day 0), the culture medium was replaced by differentiation inducer medium (DIM), consisting of DMEM supplemented with 10% fetal bovine serum (FBS) (Gibco, Cat. No. 12657), 1 μM dexamethasone (Sigma-Aldrich, St. Louis, MO, USA, Cat. No. D4902), 0.5 mM 3-isobutyl-1-methylxanthine (IBMX) (Sigma, Cat. No. 15879) and 1.67 μM bovine insulin (Sigma, Cat. No. 16634). From the second day of differentiation, the cells were maintained in culture medium containing only 0.83 μM insulin and 10% FBS which was changed every 48 hours for eight days (Alonso-Vale et al., 2009). The co-culture formed with LLC (LL/2 LLC1 - ATCC^®^ CRL-1642™) tumor cells (c/c-LLC) was introduced to the preadipocyte 3T3-L1 culture on day-2 of differentiation. The LLC cells were plated at 1 × 10^4^ on Transwell filters (Corning Inc, Corning, NY, USA, Cat. No. 3415) with 2 μm pore, therefore, without direct contact between the LLC and the preadipocytes, only shared the same culture medium. The co-cultures were maintained at 37 °C with 5% CO_2_ (Ailhaud, 2001). A photographic record was conducted on days 0, 2, 4 and 8 of differentiation. At the last day of the differentiation, the protocol was adopted staining by Oil Red-O.

### 2.2 Oil Red-O staining

After differentiation protocol, lipid levels of newly differentiated adipocytes were quantified using the Oil Red-O staining (Amresco, Solon, OH, USA, Cat. No. 0684). Cells were washed with phosphate-buffered saline (PBS), fixed in 10% formalin for 30 minutes at 37 °C; the cells were washed with water and incubated for 5 minutes with 60% isopropanol. Subsequently, the isopropanol was discarded and the cells were incubated for 5 minutes with a solution of Oil-Red-O. The stained cultures were washed several times with water to be removed from all dye residue; stained cells were visualized by light microscopy with the photographic record, then the triacylglycerol (TAG) colored in red was quantified at 490 nm in Synergy H1 (Biotek, Winooski, VT, EUA).

### 2.3 Gene expression analyses

Total RNA was extracted and purified from 3T3-L1 samples using the RNeasy Mini Kit (Qiagen, Hilden, Germany Cat. No. 74104) following manufacturer's recommendations. RNA concentrations were determined by measuring the absorbance of 260/280 nm. Total RNA was used at 1 μg reaction containing oligodT (500 μg/ml), 10 mM of each dNTP, 5X First-Strand Buffer, DTT, and 200 U reverse transcriptase (SuperScript II, Invitrogen). Gene expression analyses were measured using SYBR green qPCR master mix (Fermentas Life Sciences, Waltham, MA, USA) as described for Batista Jr et al (Batista et al., 2012a). Primer sets for mouse preadipocyte factor 1 (Pref-1) (NM_001190705.1: sense: GGG AGA ACC ATT GAT CAC G and antisense: ACA ATG GAA CTT GCG TGG AC), CCAAT enhancer binding protein beta (C/EBPβ) (NM_001287738.1: sense: GCG CCG CCT TAT AAA CCT and antisense: GCC ACT TCC ATG GGT CTA AA), CCAAT enhancer binding protein alpha (C/EBPα) (NM_001287514.1: sense: TCC CGG GTA GTC AAA GTC AC and antisense: CCT TCA ACG ACG AGT TCC TG), peroxisome proliferator-activated receptor gamma (PPARγ) (NM_001308354.1: sense: CAC CTC TTT GCT CTG CTC CT and antisense: AGA CAA CGG ACA AAT CAC CA), fatty acid-binding protein 4 (FABP4) (NM_024406.2: sense: CTT GTG GAA GTC ACG CCT TT and antisense: CTG GTG CAG GTG CAG AAG T), perilipin (NM_001113471.1: sense: CGT GCT CAG AGA GGT TAC AGC and antisense: CAC TGC GGA GAT GGT GTT C), adiponectin (NM_009605.4: sense: TGT CTG TAC GAT TGT CAG TGG A and antisense: TAA CGT CAT CTT CGG CAT GA). The results for mRNA concentrations are expressed as a ratio over ribosomal protein L19 (RPL-19), which was amplified as a housekeeping gene using the following primers: RPL-19 (NM_001159483.1: sense: CTG ATC AAG GAT GGG CTG AT and antisense: ACC CTT CCT CTT CCC TAT GC).

### 2.4 Western blot analyses

The protein samples were extracted and purified through the speaker system 500 Vivaspin MWCO PES (GE Healthcare, Piscataway, NJ, USA, Cat. No. 28-9322-37). Western blot analyses were performed as described (Batista et al., 2012a). Primary antibodies against caspase-3 (at 1:1000 dilution) (Abcam, Milton, Cambridge, UK. Cat. No, ab44976), hormone-sensitive lipase (HSL) (at 1:4000 dilution) (Abcam Cat. No. ab45422) and adipose triglyceride lipase (ATGL) (at 1:2000 dilution) (Cell Signaling, Beverly, MA, USA, Cat. No, 2138S). All primary antibody incubations were made overnight at 4 °C and secondary anti-rabbit IgG conjugated to HRP (at 1:3000 dilution) (Cell Signaling, Cat. No. 7074) for 1 hour at room temperature.

### 2.5 ELISA

Cytokines IL-6 (M6000B), TNF-α (MTA00B) and adiponectin (MRP300) levels in the culture medium were detected by commercial cell culture kit by enzymatic-colorimetric ELISA according to the manufacturer's instructions (R&D Systems, Minneapolis, MN, USA).

### 2.6 Statistical analyses

Data analysis was performed using GraphPad Prism software 6 (GraphPad, San Diego, CA, USA). The mean and ± standard error (SE) of the mean were calculated for all variables used. We used ANOVA two-way followed by Tukey's post-test for comparison of more than 2 averaged over time; the significance adopted was p < 0.05.

## 3 Results

### 3.1 LLC co-culture system reduces adipogenesis

**Fig. 1:**
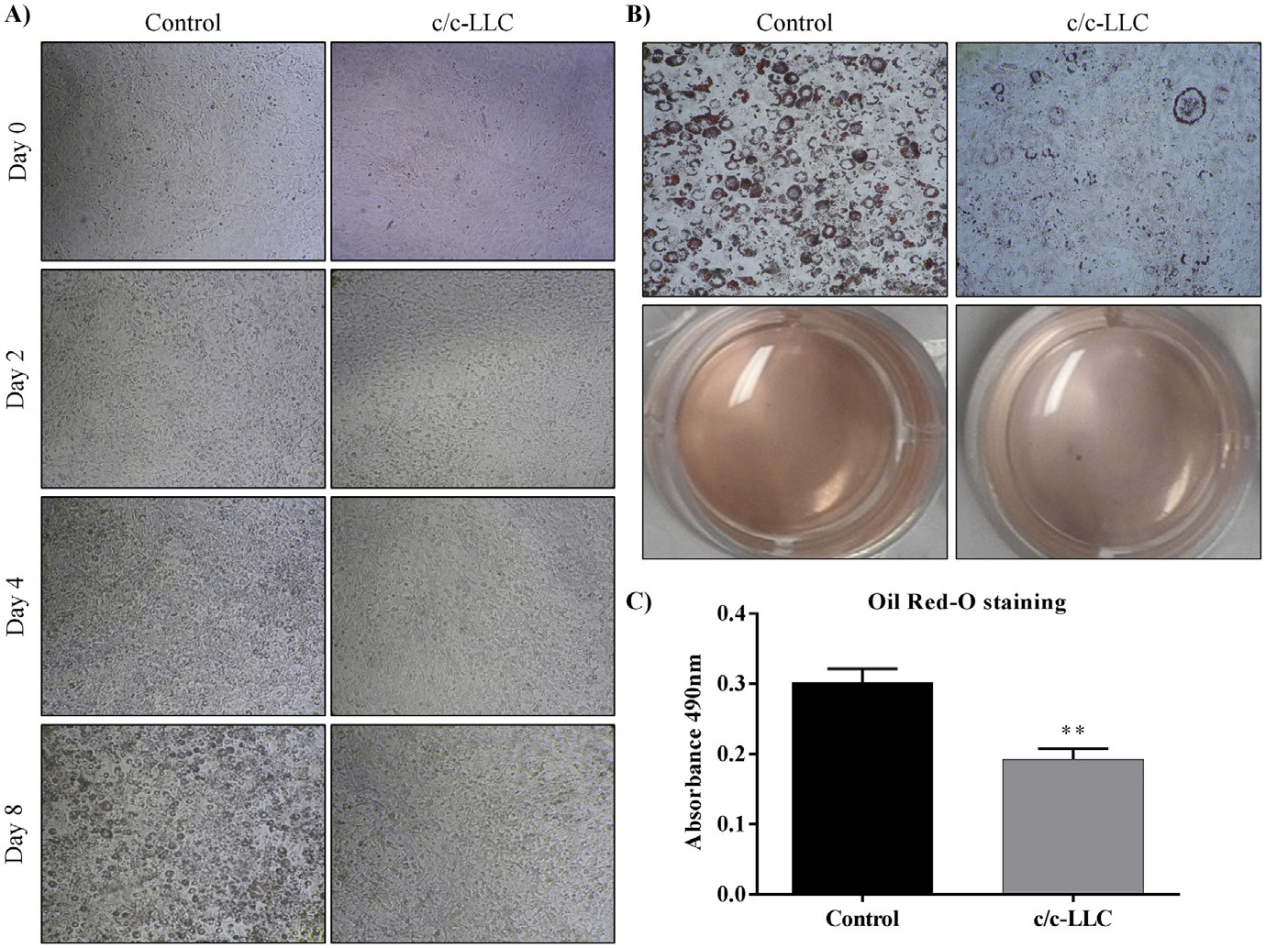
Adipocyte differentiation in 3T3-L1 cells. (A) recording of the adipogenic period from day 0 to day 8 of 3T3-L1 cells in co-culture with LLC cells (c/c-LLC). (B) The lipid accumulation in cells in c/c-LLC on day 8 using Oil Red-O staining. (C) Quantification of Oil Red-O staining by absorbance (490 nm) of the cells in c/c-LLC. The data shown are the mean ± SE (n = 4). *p < 0.05.

3T3-L1 preadipocytes were grown to confluence, exposed to c/c-LLC (day -2) and two days later (day 0) both cells were exposed to DIM. Later, we analyzed temporally (days 0, 2, 4 and 8) the differentiation potential of preadipocyte 3T3-L1 cells by inducing them in the appropriate media into adipocytes. DIM-induced the adipogenic process in 3T3-L1 as expected (Ailhaud, 2001; Alonso-Vale et al., 2009; Vernochet et al., 2009) and cells showed a progressive morphological change in the co-culture system (Fig. 1 A). Differentiated cells stained positive with Oil Red-O stain at day 8 (Fig. 1 B); the concentration of dye present in TAG accumulation of adipocytes were evaluated. At the end of the adipogenic protocol (day 8), there was a decreased of 36.2% (*p* = 0.004) in lipid accumulation in c/c-LLC cells when compared to controls (Fig. 1 C). Data showed that c/c-LLC were sufficient to inhibit the cellular differentiation process.

### 3.2 LLC affects the intermediary/late genes related to adipogenic process

**Fig. 2:**
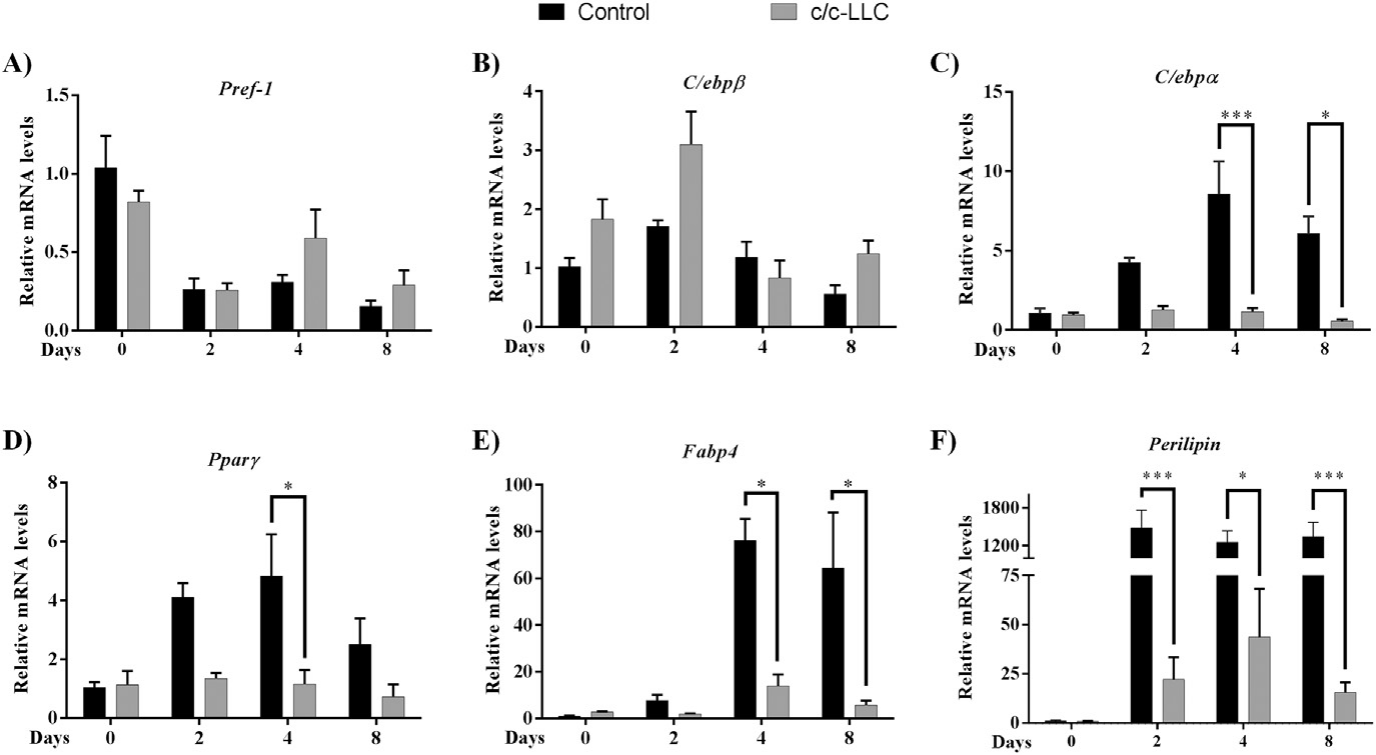
The gene expression of adipogenic factors in 3T3-L1 cells. The gene transcript levels of *Pref-1* (A), *C/ebpβ* (B), *C/ebpα* (C), *Pparγ* (D), *Fabp4* (E) and *Perilipin* (F) in cells c/c-LLC on days 0, 2, 4 and 8 were examined by RT-qPCR normalized to *Rpl-19* levels. The data shown are the mean ± SE (n = 3). *p < 0.05; **p < 0.001; ***p < 0.0001.

**Fig. 3:**
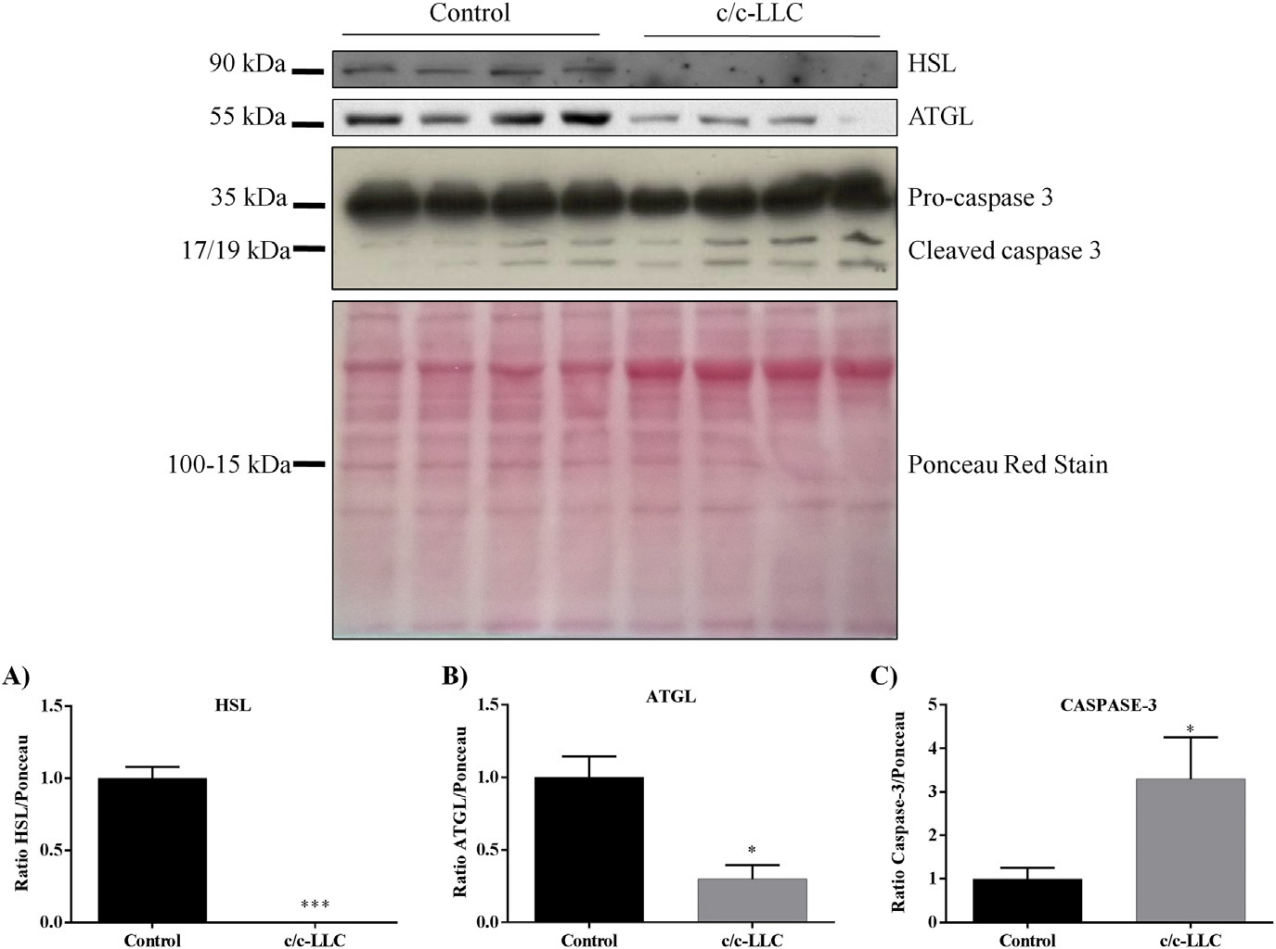
The protein expression in 3T3-L1 cells. Western blot analyses of HSL (A), ATGL (B) and caspase-3 (C) in cells c/c-LLC on day eight normalized to Ponceau Red. The data shown are the mean ± SE (n = 4). *p < 0.05; **p < 0.001; ***p < 0.0001. Full sized western blot images are shown in Supplementary Fig. 1.

To evaluate the main genes that participate in the adipogenic process during the development of cellular differentiation, we determined the mRNA expression on days 0, 2, 4 and 8 of several genes related to adipocyte phenotype such as Pref-1 (a marker of non differentiated adipocyte), C/EBPβ (adipogenic protein related to early adipogenesis), PPARγ and C/EBPα (responsible for differentiation and maturation of adipocytes), and FABP4, perilipin, and adiponectin (mature adipocyte phenotype markers). Our data show that the c/c-LLC had an inhibitory effect on the expression of key genes involved in cell differentiation, *i.e*., PPARγ, C/EBPα, perilipin and FABP4 (Fig. 2 C–F). Interestingly, there was a reduction in the expression of PPARγ (76.1%, *p = 0.02*) on day 4 (Fig. 2 D) and C/EBPα on day 4 (86.6%, *p = 0.0003*) and day 8 (90.9% *p = 0.005*) of the differentiation period in their respective controls (Fig. 2 C). Regarding the genes related to mature phenotype, perilipin expression showed downregulation of 98.4% on day 2 (*p < 0.001*), day 4, 96.5% (*p = 0.0004*) and day 8, 98.8% (*p = 0.0001*) (Fig. 2 F) and, FABP4 downregulated 81.6% (*p = 0.004*) on day 4 and 91% (*p = 0.0068*) on day 8 (Fig. 2 E), both compared to their respective controls. These data suggest the products secreted by c/c-LLC may affect intermediary and late genes expression during adipogenesis. The expression of C/EBPβ, did not have statistical significance between the c/c-LLC and control groups. The expression of Pref-1, a known repressor of adipocyte differentiation (O'Connell et al., 2011), also had no statistical significance between the groups c/c-LLC and controls. Furthermore, we have identified that adipocytes in c/c-LLC presented a decreased protein expression of key ATGL (70%, *p = 0,006*) and HSL (100%, *p < 0.0001*) (Fig 3 A–B).

### 3.3 C/c-LLC induces apoptosis in 3T3-L1

At the end of the cell differentiation protocol, we evaluated the expression levels of one the proteins participating in the cell death signaling pathway, caspase-3. On day eight there was a trend toward to increase of 2.2-fold, (*p = 0.05*) in the cleaved form of the protein (Fig. 3 C), suggesting that c/c-LLC not only inhibits adipogenesis but also stimulates apoptosis, possible compromising AT turnover.

### 3.4 C/c-LLC increases the secretion of inflammatory factors

**Fig. 4:**
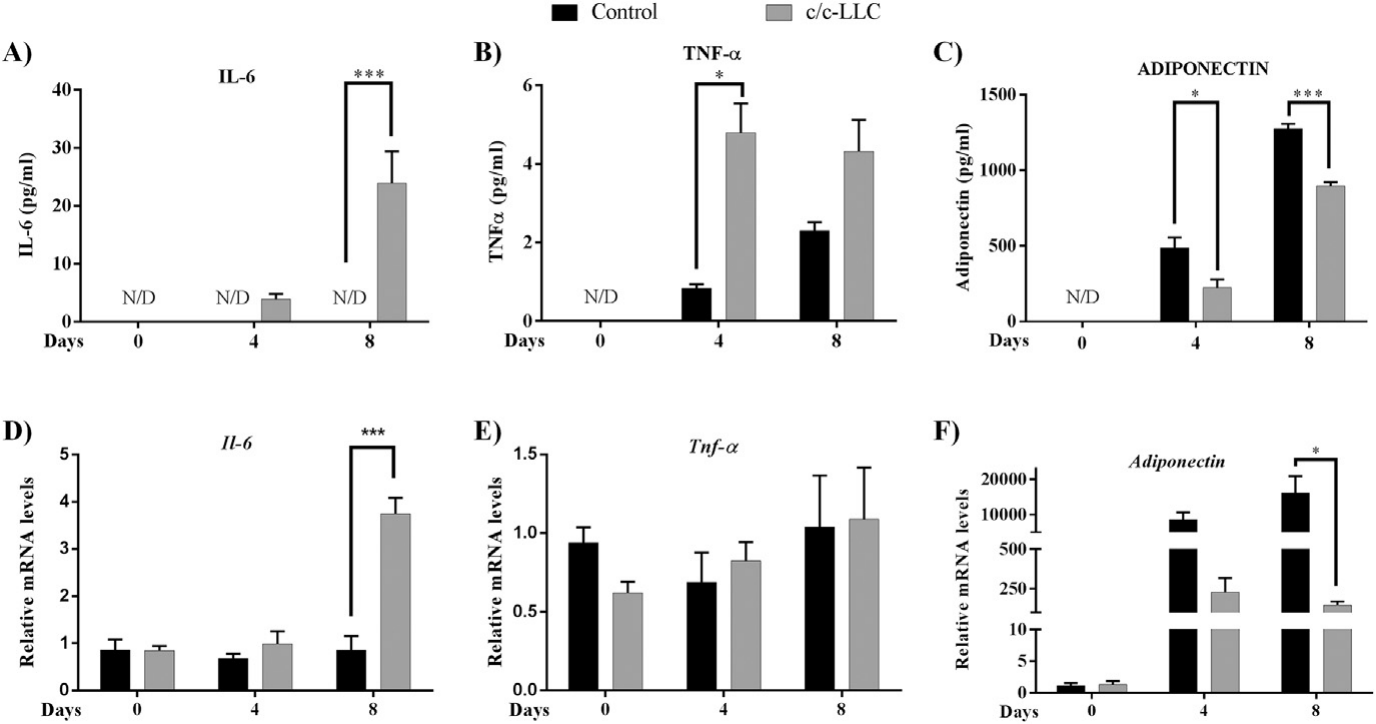
The factors secreted by cells 3T3-L1 and LLC. Culture medium protein analyses of IL-6 (A), TNF-α (B) and ADIPONECTIN (C) were examined by ELISA and gene expression levels of *Il-6* (D), *Tnf-α* (E) and *Adiponectin* (F) were examined by RT-qPCR normalized to *Rpl-19* levels. All the groups were examined on days 0, 4 and 8. The data shown are the mean ± SE (n = 3). N/D (non-detect data), *p < 0.05; **p < 0.001; ***p < 0.0001.

The next step was to analyze temporally pro-inflammatory secretion in the culture medium during the adipogenic protocol with c/c-LLC. Thus, we assessed on days 0, 4 and 8, the main cytokines present in the CC syndrome, such as TNF-α and IL-6. During the adipogenic process, the levels of TNF-α in culture medium showed large increased from day 4 (4.7-fold, *p = 0.0007*) when compared to their respective control (Fig. 4 B). However, there was no difference in the preadipocyte TNF-α gene expression between control and c/c-LLC during differentiation period (Fig. 4 E). The secretion of IL-6 showed an increase from day 8 (*p < 0.0001*) when compared to respective control (Fig. 4 A). IL-6 gene expression in the preadipocytes from c/c-LLC showed upregulation observed on the eighth day of differentiation (Fig. 4 D). Adiponectin was also evaluated in the culture medium, showing decreased secretion in c/c-LLC on fourth and eighth day, 57.1% (*p < 0.003*) and 20.9% (*p < 0.0001*) respectively (Fig. 4 C). Gene expression of the adiponectin showed downregulation from day 8 (*p = 0.0003*) when compared to controls (Fig. 4 F).

## 4 Discussion

The downregulation of adipogenic factors and possible impairment of adipogenesis in response to the presence of CC has been recently reported (Bing et al., 2006; Batista et al., 2013). In this regard, despite adipocyte atrophy, and AT remodeling are well characterized in cachexia (Bing et al., 2006; Batista et al., 2016), there is no consensus if such effects induced by cachexia would be a result of secreted products directly by the tumor and tumor × host relation. Thus, we have analyzed *in vitro* adipogenesis in a co-culture system to mimic the effects of CC on adipocytes. In the present study, co-culture of LLC promoted a decreased volume of the lipid droplets in 3T3-L1 cells, compromising its maturation process (adipogenesis) *in vitro*. This result was followed by downregulation of adipogenic and lipolytic genes expression, increased in apoptosis markers and proinflammatory cytokines secretion by both tumor cells and adipocytes. In this sense, taken together, these data suggest that the presence of the tumor cells were able to inhibit the adipocytes maturation with was associated with the increased levels of inflammatory cytokines.

In a co-culture system, the adipogenesis induced in 3T3-L1 cells was categorically inhibited. As far as we know, this was the first study that showed this effect directly induced by LLC cells. Also, we evaluated Pref-1 gene expression to label the pre-adipocytes not compromised with adipogenesis (O'Connell et al., 2011). In this way, we demonstrated that both analyzed condition (with or without LLC) suspended the expression of Pref-1 during the development of differentiation, indicating that both conditions were committed to adipogenesis, with no effect of LLC. C/EBPβ is a well-known trigger of early adipogenesis being rapidly expressed in the first 4 hours after induction and responsible for driving C/EBPα and PPARγ expression (Prusty et al., 2002; Farmer, 2005). In our co-culture system, C/EBPβ expression in differentiating adipocytes was not altered when compared to their respective controls, indicating that the tumor cell did not influence the early phase of differentiation. On the other hand, the data presented here differ with those observed in the animal model. Bing et al. (2006), showed a reduction of C/EBPβ expression in mice bearing a MAC16 tumor. According to the authors, the reduction of CCAAT/enhancer-binding protein and PPARγ family proteins impair the maintenance of the mature adipocyte phenotype, thereby aggravating the cachectic condition (Bing et al., 2006). The PPARγ and C/EBPα factors are described as key regulators of adipogenesis since they participate in the expression of the other proteins responsible for the mature adipocyte phenotype (Cristancho and Lazar, 2011). Temporally, C/EBPβ is directly linked to the expression of C/EBPα, in which it participates in the expression of PPARγ remaining self-regulated until the end of differentiation (Cristancho and Lazar, 2011). In the present study, inhibition of PPARγ and C/EBPα was observed in the presence of LLC from the fourth day of differentiation, which corroborates the down-regulation of these proteins observed in AT from animal (*in vivo*) models of induced cachexia (Bing et al., 2006; Batista et al., 2012a; Tsoli et al., 2014). In addition, we show that such impairment occurs in intermediate-late events of differentiation, not at the early events.

Seeking for further information regarding on adipogenesis inhibition, we have analyzed some mature adipocytes markers, such as; FABP4, perilipin, HSL, and ATGL. All those markers showed reduced protein expression throughout the differentiation period, which demonstrate the inhibition of the adipocyte maturing process by LLC. These data suggest that the reduction of fat droplets observed in adipocytes is due to the non-maturation of the adipocyte, and not through the process of increased lipolysis, which is considered to be one of the main factors responsible for the reduction of AT (Ryden et al., 2008; Tsoli et al., 2016).

The maintenance of cellularity (or cell viability) was also addressed and c/c-LLC induced increase in both total and cleavage of the caspase-3 protein, suggesting increased apoptosis. Interestingly, in Walker-256 tumor-bearing rats, a reduction of the Pref-1/Adiponectin ratio and an increase in the activation of caspase-3 were demonstrating in retroperitoneal AT, suggesting an impairment of the cellular turnover induced by CC. However, further studies should be performed actually to check for apoptosis pathway modulation in adipocytes during CC.

Studies that emphasize the inhibition of the adipogenic process by-products secreted by tumor cells have demonstrated that such effect may occur due to the release of exosomes derived from tumor cells (Wang et al., 2017). As well as the secretion of pro-inflammatory cytokines such as TNF-α, which is described by inhibiting the adipogenic process by inhibiting PPARγ expression (Meng et al., 2001; Ruan et al., 2002). Furthermore, considering that cell differentiation and apoptosis process could be controlled by proinflammatory cytokines (Coppack, 2001), the next step was to evaluate the main proinflammatory cytokines described as responsible for the depletion of AT during cancer cachexia, in particular, IL-6 and TNF-α (Batista et al., 2013; Tsoli and Robertson, 2013). In cachexia induced tumor-bearing mice (LLC cells), both cytokines are increased in plasma levels (data not shown). Thus, to verify the possible origin of IL-6 and TNF-α secretion in the culture medium (adipocyte or LLC), the gene expression and cytokines secretion (culture medium) from adipocytes was evaluated during cellular differentiation. IL-6 gene expression was performed at different set points, together with the quantification of its secretion. Both results indicated high levels of this cytokine from mature adipocytes, corroborating the hypothesis that the adipose cell is an essential source of IL-6. Interestingly, previous results from our group have demonstrated that subcutaneous adipose tissue is an important source of IL-6 in cancer patients with cachexia. Moreover, this cytokine has a positive correlation with mass body reduction and tumor staging (Batista et al., 2013).

Differently from the IL-6 response, although TNF-α secretion was increased in the culture medium, TNF-α gene expression in adipocytes was not modified during adipogenesis. Interestingly, the data suggest that TNF-α secretion is primarily performed by LLC, not by adipose cells. It is known that LLC is a potent producer of TNF-α (Kim et al., 2009) and other studies emphasize its inhibitory role in adipocyte maturation through the negative regulation of PPARγ and C/EBPα expression (Warne, 2003; Cawthorn et al., 2007). According to Cawthorn et al. (2007), the antiadipogenic action of TNF-α occurs through the activation of tumor necrosis factor receptor 1-mediated death domain where it increases the activity of TCF4-dependent transcriptional and stabilization of beta-catenin, which in turn inhibits the expression of PPARγ and C/EBPα in 3T3-L1 cells (Cawthorn et al., 2007). In cancer cachexia patients, these circulating levels of TNF-α are elevated, but not correlated with clinical markers of cachexia (Batista et al., 2013). In this study, we did not evaluate the involvement of pathways involved in the inhibition process, nor even the participation of other inflammatory cytokines expressed by the tumor cell or present in the cachexia.

## 5 Conclusion

In summary, our study has shown the inhibition of the adipogenic process in a 3T3-L1 co-culture system with LLC cells, which occurred in parallel with the increase of inflammatory cytokines (TNF-α and IL-6) present in the culture medium. The secretion of IL-6 showed the contribution from adipocytes. TNF-α was predominantly produced by tumor cells, suggesting the participation of both cell types in the secretion of inflammatory cytokines into the culture medium. Finally, these results reinforce the importance of a better understanding of the interaction between the host and tumor for the study of cancer cachexia.

## Declarations

### Author contribution statement

Magno Alves Lopes: Conceived and designed the experiments; Performed the experiments; Analyzed and interpreted the data; Wrote the paper.

Felipe Oliveira Franco, Felipe Henriques: Performed the experiments.

Sidney Barnabé Peres: Analyzed and interpreted the data.

Miguel Luiz Batista Jr.: Conceived and designed the experiments; Analyzed and interpreted the data; Contributed reagents, materials, analysis tools or data; Wrote the paper.

### Funding statement

This work was supported by São Paulo Research Foundation (FAPESP) Grants: 2010/51078-1, 2015/19259-0 and CNPq 311966/2015-2 to M.L.B.Jr. The contents of this article are solely the responsibility of the authors and do not necessarily represent the official views of FAPESP.

### Competing interest statement

The authors declare no conflict of interest.

### Additional information

No additional information is available for this paper.
