# Supplementary figures and images for: LLC tumor cells-derivated factors reduces adipogenesis in co-culture system
Source: Heliyon. 2018 Jul 30;4(7):e00708. doi: 10.1016/j.heliyon.2018.e00708 (PMC6071679; doi:10.1016/j.heliyon.2018.e00708)

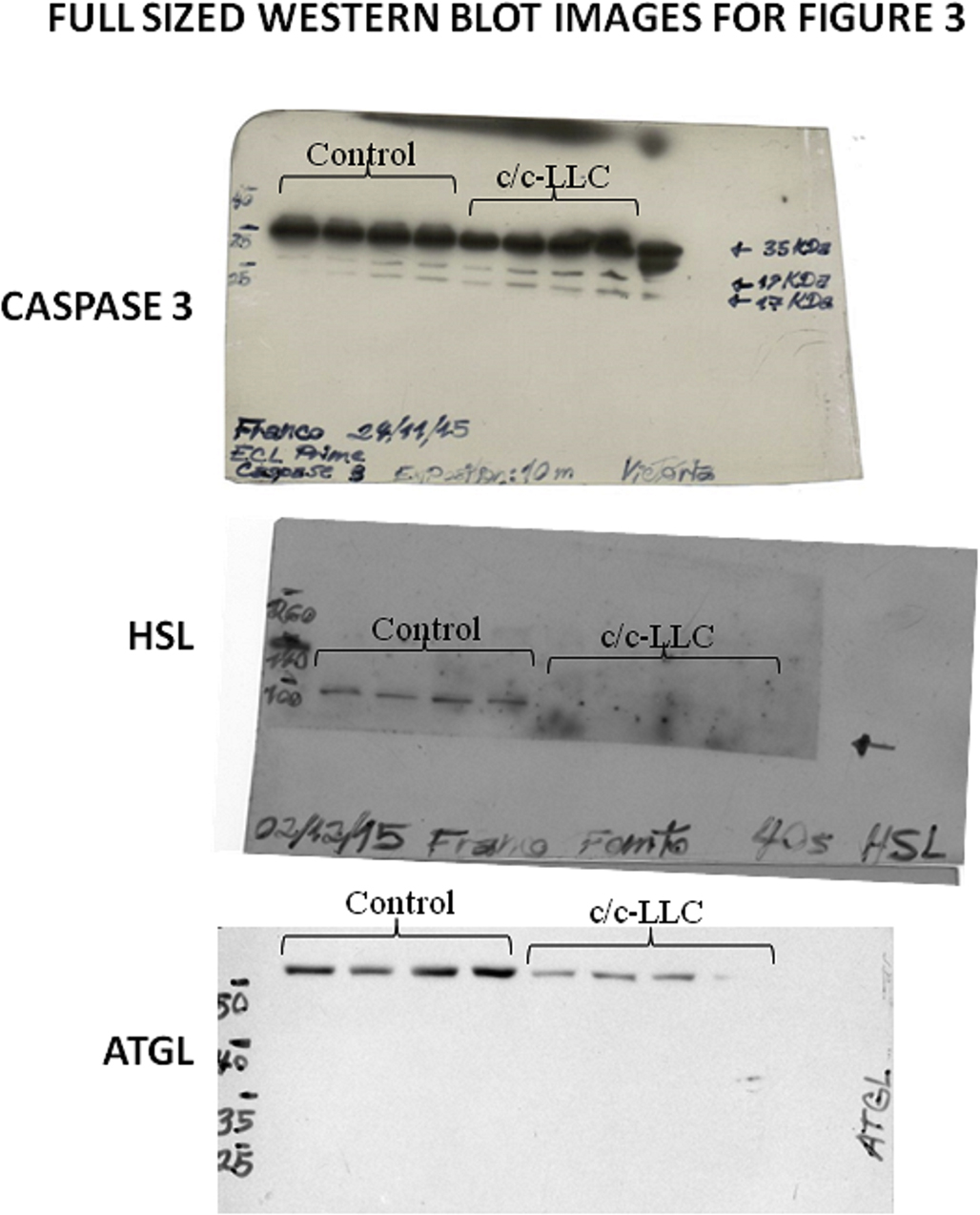

Supplement: Supplementary Figure 1 [file figs1.jpg]
